# Digestive Potential of Soybean Agro-Industry Byproducts

**DOI:** 10.3390/ani10050911

**Published:** 2020-05-25

**Authors:** Fagton Negrão, Clayton Dantas, Anderson Zanine, Daniele Ferreira, Marinaldo Ribeiro, Alexandre Souza, Michelle Parente, Henrique Parente, Ivo Cunha, Thiago Nascimento, Anny Lima, Cledson Sá, Danrley Bandeira

**Affiliations:** 1 Federal Institute of Education, Science and Technology of Rondônia, Colorado do Oeste RO 76993-000, Brazil; fagton.negrao@ifro.edu.br; 2 Federal University of Mato Grosso, Rondonópolis MT 78735-901, Brazil; claytonagm@zipmail.com.br (C.D.); alexandre@ufmt.br (A.S.); 3 Federal University of Maranhão, Chapadinha MA 65500-000, Brazil; dany_dosanjos@yahoo.com.br (D.F.); michellemrn14@gmail.com (M.P.); hnparente@hotmail.com (H.P.); ivoleme@gmail.com (I.C.); thiagovcn_vet@hotmail.com (T.N.); annygraycy@hotmail.com (A.L.); cledsongom@gmail.com (C.S.); danrleymartins12@gmail.com (D.B.); 4 Federal University of Goiás, Goiânia GO 74690-900, Brazil; malldorr@gmail.com

**Keywords:** forage conservation, *Brachiaria decumbens*, rumen liquid, ruminal parameters, ruminant

## Abstract

**Simple Summary:**

This manuscript addresses the use of nutritional alternatives: that the addition of soybean hull levels affect protein and carbohydrate fractionation, with significant changes in rumen degradability; that most of the analyzed variables were affected linearly, so levels of additive inclusion resulted in the satisfactory growth of rumen microorganisms and synchronism between the protein and carbohydrate digestion rates; and that these had an important effect on the end products of fermentation and on animal production. This is significant because animals fed diets showing these characteristics can better express their animal performance. The results obtained in this study indicate that the addition of intermediate levels of soybean hulls close to 20–30% ensures better characteristics in the silage of *Brachiaria decumbens*.

**Abstract:**

This study aimed to determine the protein and carbohydrate fractions as well as the in situ rumen degradability of *Brachiaria decumbens* silage (BDS) supplemented with soybean hulls. Five soybean hull inclusion levels were used: 0, 10, 20, 30, and 40% of the fresh matter of *B. decumbens* grass, distributed into a completely randomized design with five replications. The inclusion of soybean hulls caused a linear decrease (*p* < 0.001) in carbohydrate fractions A + B1 and a linear increase (*p* < 0.001) in carbohydrate fraction C. The percentage of non-protein nitrogen fraction increased linearly (*p* < 0.001), but the nitrogen fractions B_1_ + B_2_ and B_3_ presented a negative quadratic effect (*p* < 0.01) with soybean hull level and fraction C presented a linear decrease (*p* < 0.001). The dry matter (DM) degradability of soluble fraction (A) and the undigestible DM decreased linearly (*p* < 0.01) with the soybean hull level. The potentially degradable water-insoluble portion (DM fraction B) and degradability rate (c) of the DM fraction B increased linearly (*p* < 0.001) with soybean hull level. The crude protein (CP) fraction A presented a linear increase (*p* < 0.001) with soybean hull inclusion; however, soybean hull levels caused a linear decrease (*p* < 0.001) in the CP level of fraction B. The degradable insoluble fraction of NDF (D) of the silage increased linearly (*p* < 0.001) and the indigestible NDF fraction of the silage was linearly decreased with the soybean hull level (*p* < 0.001). The inclusion of intermediate levels (20–30%) of soybean hulls provided better protein and carbohydrate fractions and better quality of BDS.

## 1. Introduction

Regions with tropical climate have many genera of grasses (*Pennisetum, Panicum*, *Cynodon*, and *Brachiaria*) that can be recommended for silage; among them, the grasses of the genus *Brachiaria* can be highlighted due to their high potential for biomass production, their adaptation to low soil fertility, and low pH tolerance [1]. However, the silage of these grasses may not result in the silage of good nutritional quality due to the high moisture content (approximately to 80%), low water-soluble carbohydrates (lower than 1% dry matter, DM), and high buffering power (greater than 20 EQ-HCl) on the 70th day, which is the best time for harvesting [1,2].

Byproducts from cereal and food processing may have characteristics that make them viable for inclusion in the grass silage process [2] such as the soybean hulls produced as a byproduct of the soybean industry and which can be considered a potential additive to be included in the grass silage process, due to its high water retention capacity, which limits the development of *Clostridium* spp. and allows a rapid drop in pH, so when included in *Brachiaria decumbens* silage (BDS), this byproduct improves the fermentation and nutritional value of the silage, despite presenting low water-soluble carbohydrates.

Therefore, it is very important to evaluate the nutrient compounds in silage containing agroindustrial byproducts, since the chemical composition and residue degradation rates differ. Through the knowledge of the protein and carbohydrate fractions of feeds, technological applications can be used for diet formulation [3,4] with the aim of improving animal performance.

Thus, given the lack of information on grass silage with the inclusion of levels of soybean hulls, the present research aimed to evaluate the fractions of carbohydrates and protein, and the in situ ruminal degradation kinetics of BDS enriched with ground soybean hulls for adult crossbreed cows (Holstein x Zebu).

## 2. Material and Methods

This study was conducted at the Federal University of Mato Grosso, in the period from November to March 2015, according to the recommendations of the Committee on the Ethics of Animal Experiments Guide of the Federal University of Mato Grosso (protocol number 23108.046399/13-4).

### 2.1. Silage Preparation

The forage used was *Brachiaria decumbens* grass (BDG) from an established pasture of approximately 0.5 ha. The grass was subjected to a standardization cut performed with a mower attached to a tractor at 5 cm above ground level. On the same day, the soil was top-dressed with 60 kg×ha^−1^ of N and 60 kg×ha^−1^ of K_2_O, using ammonium sulfate and potassium chloride, respectively. After 60 days of regrowth, the grass was harvested mechanically at 10 cm above ground level and chopped to a theoretical length of 3 cm using a stationary chopper (Nogueira EN-6500). At this stage, the grass presented an average height of 70 cm.

Treatments consisted of BDG (alone) and BDG with the inclusion of 10, 20, 30, or 40% soybean hulls, based on fresh matter. Immediately before ensiling, the soybean hulls were mixed according to the level established for each treatment.

The BDG was ensiled in 10 L experimental bucket silos after adding the various levels of soybean hulls. The 25 silos, five for each level, were sealed with adhesive tape, weighed, and stored in a covered area at room temperature.

### 2.2. Sample Collection and Analyses

After 45 d of ensiling, each silo was opened and its respective silage mixed according to the levels of soybean hulls. For chemical analysis (Table 1), samples of fresh BDG and its silage were dried in a forced-air oven at 55 °C for 72 h, and then ground in a Willey mill (TE-625, TECNAL, Piracicaba, São Paulo, Brazil) through 1-mm and 2-mm sieves. The concentrations of DM (method 934.01), ash (method 924.05), crude protein (CP; method 976.06), acid detergent fiber (ADF; method 973.18), and ether extract (EE; method 945.16) were determined as described in [5]. Neutral detergent fiber (NDF), cellulose, hemicellulose (HEM), and lignin were determined as described in [6].

The pH was analyzed by [7]. Organic acid analyses, according to [8], and the losses were analyzed by the methodology in [9] (Table 2).

### 2.3. Carbohydrate Fractions (A + B1, B, and C)

Carbohydrate fractions A + B1 are soluble sugars, starch, and pectin; fraction B2 (cellulose and hemicellulose) corresponds to the potentially digestible neutral detergent fiber (NDF); and fraction C is represented by the ADF.

The percentage of total carbohydrates (TC) was obtained according to [10] by the equation: TC = 100 − (%CP + %EE + %ash). Non-fibrous carbohydrates (NFC), which correspond to the fractions A + B1, were calculated by the difference between TC and NDF corrected for ash and protein (NDFap), according to [11]. Fraction C, which corresponds to the indigestible neutral detergent fiber (INDF), was calculated according to [12]. Fraction B2, which is the available fraction of the fiber, was obtained by the difference between NDFap and fraction C.

### 2.4. Protein Fractions (NPN, B1 + B2, B3, and C)

The non-protein nitrogen (NPN) fraction is soluble in the rumen and was calculated according to [13]; fraction B represents the true protein, which is subdivided into three subfractions based on the ruminal degradation speed: B1 is the fraction rapidly degraded in the rumen (>50%/hour) represented by albumins and globulin; and B2 is the fraction with an intermediate degradation rate (5–15%/hour). These fractions, B1 + B2, which were obtained as reported by [14], represented the fraction of effectively degraded proteins and unbound fiber. Fraction B3 was obtained by the difference between the Neutral Detergent Insoluble Nitrogen (NDIN) and Acid Detergent Insoluble Nitrogen (ADIN) contents. Fraction C corresponded to ADIN. The NDIN and ADIN contents were obtained according to [10].

### 2.5. Degradability and Sample Analysis

For the in situ assay, four non-lactating, non-pregnant, rumen-fistulated crossbred cows (Holstein × Zebu) with an average body weight of 400 kg were maintained on a *B. decumbens* pasture and received mineral supplement ad libitum at the trough. The BDS was ground to 2 mm, weighed, and placed in non-woven textile (TNT) bags to provide 20 mg of the sample, according to the method of [15]. Bags were heat-sealed and conditioned in a shade-tissue bag with 100 g of lead (to prevent bags from remaining only at the dorsal part of the rumen), tied with a nylon wire, and inserted in the rumen for 0, 2, 4, 8, 16, 24, 48, 72, 96, and 144 h. The cows were incubated twice, totaling eight replications per level for each hour. The bags of time 0 h were removed approximately 5 min after incubation. After incubation, bags were removed from the rumen at the same time and washed in running water for approximately 30 min to remove the excess rumen content [16]. Subsequently, the bags were washed exhaustively in running water until clear and then sent to the Laboratory of Animal Nutrition, where they were dried in a forced ventilation oven at 65 °C for 48 h, according to [5]. The residue obtained after this step was used for analysis.

The in situ rumen degradation data of DM, CP, NDF, and INDF were calculated as the difference found for each component between the weights before and after rumen incubation, expressed as a percentage of the original material in the bags, so it was possible to build rumen degradation profiles. To estimate the rumen degradation kinetic parameters of DM and CP, the first-order asymptotic exponential model was used according to [17], and for the NDF degradation profile, the model of [18] was adopted. Using these models for the DM and CP contents, the following components were estimated: fractions A (soluble fraction), B (potentially degradable insoluble fraction), c (ruminal degradation rate of fraction B, expressed in percentage per hour); and I (indegradable fraction). For the NDF, fractions D (degradable fraction), *d* (ruminal degradation rate of fraction D, expressed in percentage per hour), and INDF (indegradable fraction) were estimated according to [17].

### 2.6. Statistical Analysis

Five soybean hull inclusion levels were tested: 0, 10, 20, 30, and 40% of the fresh matter, distributed into a completely randomized design with five repetitions per soybean hull inclusion level.

The degradability profiles for DM, CP, and NDF were subjected to adjustment by the respective models, utilizing the procedure in [19]. The effect of the soybean hull inclusion levels was evaluated using PROC Mixed, according to the following model:

Yij = μ + Ti_*i*_ + *eij*

where Yij is the dependent variable; μ is the overall mean; Ti is the fixed effect of soybean hulls inclusion levels 0, 10, 20, 30, and 40%; and *eij* is the residual error.

Polynomial orthogonal contrasts were used to test the linear and quadratic effects of the soybean hull inclusion levels at 0, 10, 20, 30, and 40% and the rumen kinetics parameters of the *B. decumbens* silage.

## 3. Results

### 3.1. Fraction of Carbohydrate and Protein

The total carbohydrates in BDS decreased linearly (*p* < 0.001) by 0.099% for each 1% of soybean hull added to the BDS (Table 3).

Soybean hull inclusion decreased linearly (*p* < 0.001) in the A + B_1_ carbohydrate fraction, causing a drop of 0.57% for each 1% inclusion of the additive. The fraction B_2_ decreased linearly (*p* < 0.001) with the soybean hull levels. The soybean hull inclusion increased linearly (*p* < 0.001) for fraction C, causing an increase of 0.54% for each 1% inclusion of the additive.

The non-protein nitrogen (NPN), which corresponds to the soluble fraction, increased linearly (*p* < 0.001) with the soybean hull levels, with an estimated increase of 0.63% for each 1% soybean hull added to the grass silage (Table 3).

The B_1_ + B_2_ concentration (% total nitrogen) showed a negative quadratic effect (*p* < 0.001) with the soybean hull levels and its minimum level was estimated at 17.21% at 24.31% soybean hull inclusion. The cell-wall-associated protein (B_3_ fraction), which showed slow rumen degradation, had a negative quadratic effect (*p* < 0.001) with the soybean hull inclusion, and its minimum concentration was estimated at 13.55% at 23.75% of soybean hull inclusion. The fraction considered undigestible (fraction C) decreased linearly (*p* < 0.001) with soybean hull inclusion.

### 3.2. Degradability

Fraction A and fraction I of DM decreased linearly (*p* < 0.001), and fraction B and the degradation rate of DM (c) increased linearly (*p* < 0.001) with the soybean hull level (Table 4).

The CP degradability of fraction A increased linearly (*p* < 0.001) with the soybean hull inclusion; however, the soybean hull inclusion caused a linear decrease (*p* < 0.001) in the CP of fraction B, fraction I, and degradation rate of CP (c; Table 4).

Soybean hull inclusion increased linearly (*p* < 0.001) in fraction D with an estimated increase of 0.53% for each 1% soybean hull added to the grass silage. The degradation rate of NDF (d) and INDF (indigestible fraction) showed a linear decrease (*p* = 0.018) with the additive inclusion in the grass silage (Table 4).

## 4. Discussion

The reduction in TC value can be related to the higher levels of CP and EE present in the silage enriched with soybean hulls (Table 1). These nutrients can interfere with the TC estimates because they are used in the equation to calculate TC [10]. The FC corresponds to the available and unavailable fiber, represented by hemicellulose and cellulose, along with lignin. With the exception of lignin, these particles are partially available in the rumen, and thus have great influence on the availability of energy for ruminants [19]. The decreases in NFC content observed in the present study can be related to the higher level of NDF in the additive (70%) in relation to the grass (59.1%) (Table 1). Feeds with a low A + B_1_ fraction may compromise the growth of some species of rumen microorganisms and the synchronism between the protein and carbohydrate digestion rates has an important effect on the final fermentation products and consequently on animal production [18].

Thus, the NDF present in soybean hulls contributed to decreasing the A + B_1_ fraction of BDS. It should be mentioned that the large variation in the concentrations and components of FC and NFC, and their respective availabilities in the rumen, might cause varied responses in animal performance, which justifies the determination of their fractions for the dietary adequacy of ruminants. The behavior presented in this study can be explained by the lower cell-wall contents of the additive in relation to BDG, as reported in the literature [20,21]. The B_2_ fraction, presenting a slow degradation rate along with fraction C (indigestible), usually affects the animal intake by the rumen fill, which can reduce animal performance [18]. The inclusion of soybean hulls caused an increase in fraction C, but the lignin in these soybean hulls had characteristics of lower complexation with cellulose and thus these interactions can cause a greater amount of degraded cellulose. This can be seen in Table 4, where the addition of the soybean hulls caused a reduction in fraction I in the DM and CP as well as a reduction of INDF [22].

The greater amount of the NPN fraction added to the lesser amount of A + B1 of the carbohydrate fraction caused by the inclusion of soybean hulls means that the rumen pH does not decrease abruptly (Table 3), thus maintaining a rumen environment that favors the growth of fibrolytic bacteria, resulting in greater NDF degradability (Table 4) and confirming earlier reports [22].

The increase in NPN fraction improved the forage quality because to meet the nutritional requirements of the rumen, microorganisms provide a nitrogen source so that ruminal bacteria can build their proteins and thus increase the growth and replication of the microrganisms that cause an increase in the efficiency of digestive processes with a greater use of fractions of slow degradation by increasing the supply of microorganisms present in the rumen [22,23].

The decrease in the DM degradability of fraction A can be attributed to the reduction in the content of non-fibrous carbohydrates and the increase in NPN with the inclusion of soybean hulls, which represents the uptake of degradable nutrients by the large microbial population present in the rumen of these animals [24]. Thus, we can state that the DM degradability of fraction B—potentially insoluble and degradable—could be a result of the increased fibrolytic microbiota levels and activity of the cell-wall polymers and the increased degradability of cellulose due to the less complex cellulose–lignin bond in the soybean hulls, thereby causing a decrease in the INDF fraction [25]. The increased indigestible fraction (I) of DM can be explained by the contents of cellulose (51.42%) and hemicellulose (19.54%) from soybean hulls, which could decrease fiber digestion and affect DM intake [26].

The reduction in the degradability rate (fraction c) of the insoluble and degradable fraction of the CP was due to the high amount of soluble nitrogen compounds remaining in the silage. However, even with a reduction in the % degradation of fraction B in the CP, the inclusion of soybean hulls promoted an increase in the amount of CP and NDF as well as an increase in the degradability of the NDF, which consequently increased the degradation of fraction B in the DM. The reduction of the indegradable fraction (I) of CP in BDS was closely related to the lower amount of fraction C in the fractionation of nitrogen in BDS (Table 3) as a result of the soybean hull inclusion levels. This greater degradability was associated with a higher amount of CP with the inclusion of soybean hulls, increasing the availability of nitrogen, which increased DM degradability by providing a greater number of added fibrolytic bacteria. Additionally, the lower lignin and cellulose binding complexity of soybean hulls caused a lower amount of fraction I in DM degradability, which can impact DM intake and the amount of energy available for the rumen microorganisms after digestion [22,24].

The increase in the degradation of the NDF (fraction D) can be explained by the low lignin content and low complexity of lignin-cellulose bound in the soybean hulls [27], which favors the action of microflora present in the ruminants’ digestive systems [22]. According to [28], the variation in the degradation rate of fraction *d* is due to the preference of rumen bacteria for different types of plant tissues. This observation is consistent with the fact that greater levels of additive, in this study, may have favored the rumen microbial population responsible for degradation of the non-structural carbohydrates present in the silage. The reduction of the INDF can be considered an advantage because a decrease in this fraction implies more energy available for the animal [12,24].

However, excess NPN degradation can be harmful to ruminants, mainly because the high ammonium nitrogen supply resulting from the accelerated rate of NPN degradation causes NH_3_–N to be transformed into urea, which is less toxic to the animal [28,29]. This urea, depending on the CP diet, can be recycled via saliva and returned to the rumen and increase the nitrogen supply during rumination or, when in excess, can be excreted via the urine or even in milk in the case of lactating females [30]. As far as the carbohydrate and nitrogen fractions and degradability parameters of BDS in this experimental situation are concerned, soybean hulls can be included at up to 40% of BDS fresh matter. However, the use of this level of inclusion combined with grass cut in an inadequate harvest time can provide inadequate fermentation and reduce animal performance.

## 5. Conclusions

The inclusion of up to 40% of soybean hulls in the fresh matter in the *Brachiaria decumbens* silage cut at 60 days improved the degradability parameters of the silage; however, as the inclusions were in the fresh matter, we recommend intermediate levels of soybean hull (20 to 30%) inclusion to ensure better characteristics in the *Brachiaria decumbens* silage regarding protein and carbohydrate fractionation and the degradability of the indigestible fractions of dry matter, crude protein, and neutral detergent fiber.

## Figures and Tables

**Table 1:** Chemical composition (DM basis) of soybean hulls, *B. decumbens* grass, and *B. decumbens* silage.

Parameters Evaluated (%DM)	Soybean Hull (%)	*B. decumbens*	Soybean Hulls Levels (%)				
			0	10	20	30	40
Dry matter ^1^	90.00	26.73	21.46	29.59	37.72	45.84	53.93
Ash	3.85	8.55	10.44	8.35	6.91	5.60	5.67
Crude protein	14.00	6.26	5.48	6.79	8.10	9.42	10.73
NDF ^2^	70.00	59.10	59.10	61.07	61.39	62.13	61.39
ADF ^3^	53.00	55.41	29.52	33.26	36.95	40.64	44.33
TDN ^4^	77.00	69.88	69.88	71.06	71.86	73.81	74.46
Ether extract	2.60	2.08	2.08	3.18	3.67	4.61	5.91
Cellulose	51.42	31.29	-	-	-	-	-
Hemicellulose	19.54	30.48	29.58	27.81	24.44	21.49	17.06
Lignin	3.43	6.53	-	-	-	-	-

^1^ % on a fresh matter basis; ^2^ Neutral detergent fiber; ^3^ Acid detergent fiber; ^4^ Total digestible nutrients.

**Table 2:** pH values, buffering power (BP), ammonia nitrogen (NH_3_–N), lactic acid (LA), acetic acid (AA), butyric acid (BA), propionic acid (PA), losses through gases (LG), losses through effluents (LE), dry matter recovery (DMR) in *B. decumbens* silage with soybean hull levels.

Variables	Soybean Hulls Levels (%)				
	0	10	20	30	40
pH	4.90	4.49	4.23	4.13	4.18
BP (EQ-HCl) ^1^	21.07	20.10	19.34	19.01	19.00
NH_3_-N (%TN) ^2^	14.28	14.13	12.50	12.19	9.55
LA (% DM)	3.18	4.82	4.35	3.89	3.55
AA (%DM)	1.539	1.223	0.997	0.971	0.901
BA (%DM)	0.098	0.079	0.072	0.081	0.083
PA (% DM)	0.059	0.047	0.043	0.049	0.050
**Silage Losses**					
LG (%DM)	8.55	7.36	6.16	4.97	3.77
LE (%DM)	12.53	8.53	4.55	0.56	0.00
DMR (%DM)	72.10	80.89	81.02	83.12	84.18

^1^ EQ-HCl—hydrochloric acid equivalent, ^2^ NH_3_-N (%TN) ammonia nitrogen as the total nitrogen percentage.

**Table 3:** Carbohydrate and nitrogen fractions of *B. decumbens* silage enriched with soybean hull.

Items	Soybean Hulls Levels (%)					SEM ^1^	Regression Equation	*p*-Value ^2^	
	0	10	20	30	40			L	Q
**Carbohydrate Fractions (%TC)**									
TC ^3^	82.00	81.63	81.23	80.35	77.68	0.341	Ŷ = 82.562 − 0.0992X	<0.001	0.003
(A + B_1_)	58.97	57.84	57.71	57.53	56.25	0.219	Ŷ = 58.805 − 0.575X	<0.001	0.804
(B_2_)	29.60	27.43	22.31	16.99	18.16	1.013	Ŷ = 29.562 − 0.333X	<0.001	<0.001
(C)	11.43	14.73	19.98	25.48	25.59	1.166	Ŷ = 11.262 + 0.392X	<0.001	<0.001
**Nitrogen Fractions (%TN) ^4^**									
NPN ^5^	27.59	35.92	44.02	50.78	51.40	1.752	Ŷ = 29.486 + 0.625X	<0.001	<0.001
(B_1_ + B_2_)	21.98	19.73	17.24	18.74	19.30	1.119	Ŷ = 21.508 − 0.350X + 0.0072X ^2^	<0.001	<0.001
B_3_	15.97	12.75	14.18	14.36	14.02	0.232	Ŷ = 15.358 − 0.152X + 0.0032X ^2^	0.946	0.008
C	34.36	31.60	24.56	16.12	15.28	2.197	Ŷ = 35.172 − 0.538X	<0.001	<0.001

^1^ SEM = standard error mean; ^2^ *p* value, L linear, and Q quadratic; ^3^ % in dry matter; ^4^ % of total nitrogen; ^5^ NPN = non-protein nitrogen.

**Table 4:** Mean values of the degradability parameters of A (soluble), B (potentially degradable insoluble), c (degradation rate of fraction B; expressed in percentage per hour), and I (indegradable) fractions of the dry matter (DM) and crude protein (CP), the neutral detergent fiber (NDF) degradability parameters of D (potentially degradable insoluble), and the d (degradation rate of fraction D; expressed in percentage per hour) of the *B. decumbens* silage enriched with soybean hulls.

Items	Soybean Hull Levels (%)					SEM ^1^	Regression Equation	*p*-Value ^2^	
	0	10	20	30	40			L	Q
**Dry Matter**									
A ^3^	22.77	22.54	22.08	19.63	18.04	0.500	Ŷ = 23.486 − 0.1237X	<0.001	<0.001
B ^4^	44.32	45.30	47.04	51.79	53.20	0.938	Ŷ = 43.482 + 0.2425X	<0.001	0.198
*c* (h^−1^) ^5^	0.066	0.089	0.123	0.165	0.199	0.0100	Ŷ = 0.062 + 0.0034X	<0.001	0.014
I^4^	32.92	32.16	30.88	28.58	28.76	0.650	Ŷ = 33.04 − 0.1191X	0.001	0.792
**Crude Protein**									
A ^3^	37.71	39.84	42.93	46.90	50.53	1.217	Ŷ = 37.042 + 0.327X	<0.001	<0.001
B ^4^	47.86	47.58	44.14	40.13	40.88	1.094	Ŷ = 48.54 − 0.2161X	<0.001	<0.001
*c* (h^−1^) ^5^	0.03	0.033	0.031	0.028	0.028	0.0005	Ŷ = 0.337 − 0.0002X	<0.001	<0.001
I ^4^	14.43	12.58	12.93	12.97	8.59	0.373	Ŷ = 13.063 − 0.0658x	0.089	0.351
**Neutral Detergent Fiber**									
D ^4^	56.30	49.37	52.69	57.69	58.69	0.734	Ŷ = 38.92 + 0.526X	<0.001	<0.001
*d* (h^−1^) ^5^	0.042	0.033	0.037	0.042	0.040	0.007	Ŷ = 0.0281 − 0.000334X	<0.001	<0.001
INDF ^6^	35.43	36.78	33.10	32.07	28.43	1.826	Ŷ = 36.904 − 0.1871X	0.018	0.649

^1^ SEM = standard error mean; ^2^ *p* value, L linear, and Q quadratic; ^3^ soluble fraction; ^4^ Potentially degradable insoluble fraction; ^5^ Rate of degradable feed through the animal digestive system; ^6^ Indigestible neutral detergent fiber (NDF).
